# Clinical Uses of Probiotics

**DOI:** 10.1097/MD.0000000000002658

**Published:** 2016-02-08

**Authors:** Saif Ul Islam

**Affiliations:** From the Rite Aid Corporation.

## Abstract

Probiotics are live nonpathogenic microorganisms. Many of these microorganisms are part of the normal human gut flora, where they live in a symbiotic relationship. Probiotics have been used to treat gastrointestinal (GI) and non-GI medical conditions. However, the data supporting their use are often conflicting, especially for non-GI-associated illnesses. The strongest evidence supporting the use of probiotics is related to the treatment of acute diarrhea and pouchitis. Atopic eczema in children and genitourinary infections are the only non-GI-related medical conditions where probiotics may have some beneficial effects. Product selection and dosing are not the same in all conditions, and the beneficial effects of each probiotic strain cannot be generalized.

The purpose of this article is to provide most recent information about probiotics and its uses. In contrast with previously published reviews on probiotics, we also discuss the composition of various products (Table 1), indications for their use (Table 2), product selection, and dosing of probiotics.

Probiotics are safe and appear to exert some beneficial effects in GI-related illnesses. The use of probiotics in non-GI illnesses is not sufficiently supported by current data.

## INTRODUCTION

The idea that the use of probiotics provides a health benefit is not new. However, in recent years, there has been significantly increased interest in the use of probiotics. According to Statista.com, sales of probiotics in the United States exceed 1.1 billion dollars in 2014; worldwide, probiotic sales constitute a 25 billion dollar market.^1^ Most consumers are convinced that probiotics work and utilize them for several different health conditions. Store shelves are full of probiotic products. Given that not all brands of probiotics are equally effective, selecting a particular brand for a particular condition is becoming increasingly confusing. Probiotics are considered a health food and are therefore not regulated by the Food and Drug Administration (FDA) as a drug. Although the supporting clinical data related to probiotics are ambiguous, primary care providers are often asked about the effectiveness and side effects of probiotics (Tables 1 and 2).

**TABLE 1:**
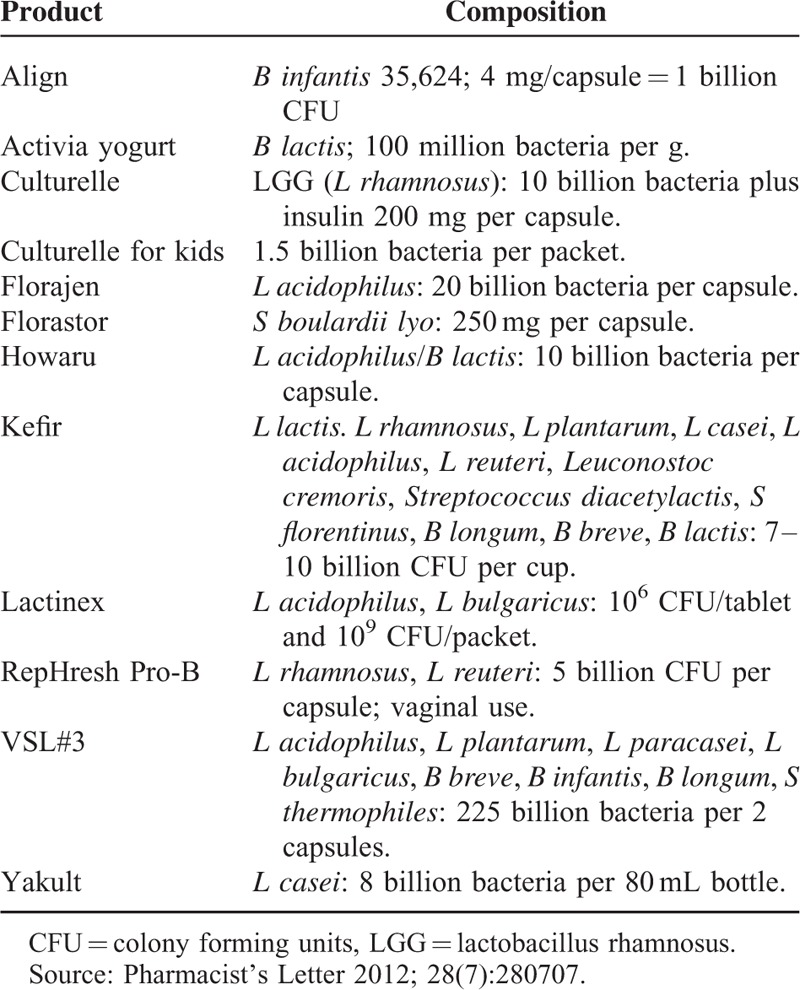
Products and Their Compositions

**TABLE 2:**
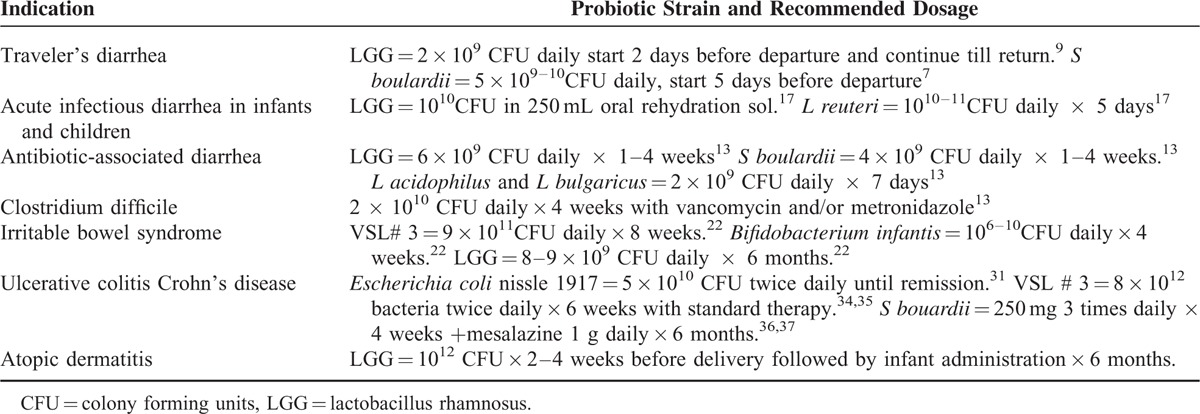
Indication, Probiotic Strains, and Recommended Dosage

## METHODS

We searched the sites below utilizing the following terms: “probiotics,” “therapeutic uses of probiotics,” “clinical uses of probiotics,” “uses of probiotics,” “Lactobacillus,” “Bifidobacterium,” “Saccharomyces,” “probiotics in travel diarrhea,” “probiotics in antibiotic-associated diarrhea,” “probiotics in IBD,” “probiotics in IBS,” “probiotics in children,” “probiotics in atopic dermatitis,” and “probiotics in Clostridium difficile.”

1. Medline
2. Cochrane Database of Systemic Review
3. American Journal of Physiology
4. Natural Medicine Comprehensive Database
5. New England Journal of Medicine
6. American Family Physician
7. American Journal of Gastroenterology
8. Journal of Pediatric Gastroenterology Nutrition
9. Clinical Pharmacology
10. Pharmacist's Letter
11. Lancet Infectious Disease
12. Clinical and Experimental Allergy
13. Natural database.com
14. BMJ

Over 100 articles and studies were reviewed. A total of 45 studies and reviews were selected for review in this article. The selection criteria included randomized, blinded studies, and unbiased articles.

No ethics committee or institutional review board was necessary because this manuscript was a systematic review and data already available in the literature. Furthermore, no experiment or study was performed on human or animal in writing this review. Therefore, no consent was needed.

## DISCUSSION

The history of probiotics goes back to centuries ago when people drank fermented milk for their health. In 1899, a scientist named Henry Tessler from the Pauster Institute in Paris discovered bifidobacterium in the intestine of breast-fed infants. He reported that infants with bifidobacterium in their intestines had fewer diarrheal episodes. However, it was a Russian scientist named Eli Metchnikoff who first proposed the idea of using probiotics for health benefits in1907. Then in 1917 a strain of *Escherichia coli* (*E coli* nissle 1917) was isolated and was used to treat patients suffering from shigellosis outbreak. Since then several others documented uses of probiotics are available in the literature, but well-designed clinical studies and data are lacking.

The term probiotic is derived from the Latin, which means “for life.” Probiotics are nonpathogenic, beneficial, live bacteria, and yeast. The most commonly used probiotics are *Lactobacillus*, *Bifidobacterium*, and *Saccharomyces boulardii*. *Lactobacillus* and *Bifidobacterium* are Gram-positive rods that are obligated facultative anaerobes and *S boulardii* is a yeast.

Lactobacillus includes several individual species, the most notable of which include *L acidophilus*, *L rhamnosus*, *L bulgaricus*, *L reuteri*, and *L casei*. Similarly, the Bifidobacterium species that are most commonly used in probiotics include *B animalis*, *B infanti*, *B lactis*, and *B longum*.

It is worth noting that not all species of probiotics are part of the normal human gut flora and that the beneficial effects attributed to 1 strain cannot be generalized to other strains.^2^

The exact mechanism of action of probiotics is unknown. However, it is proposed that several mechanisms are involved. When ingested orally, probiotics pass through the stomach and attach to the intestinal mucosa preventing epithelial attachment of pathogenic bacteria.^2–4^ Some bacteria especially lactobacillus and Bifidobacterium produce lactic acid, acetic acid, and propionic acid.^5^ These compounds lower the pH and inhibit the growth of pathogenic bacteria. Another proposed mechanism of action is their immunomodulating effects.^4^ A recent research study from the University of Maryland School of Medicine found that ingestion of LGG facilitate and modifying the activity of other bacteria, affecting the “ecosystem” of the gut.^6^

Consumers use probiotics to treat several health conditions, ranging from GI illnesses to weight loss. Many of the claims made by probiotic manufacturers are not supported by clinical data and require further studies. Moreover, there are no available sets of recommendations or FDA guidelines for probiotics. The most common use of probiotics is for gastrointestinal (GI) tract illnesses. However, it is unclear whether other illnesses that are not associated with the GI tract could benefit from the use of probiotics.

### Traveler's Diarrhea

Approximately 50% to 80% of traveler's diarrhea cases are caused by bacteria, whereas the remaining cases are caused by viruses and protozoa. *E coli* is the most common cause of bacterial traveler's diarrhea. Clinical studies have shown inconsistent results in the use of probiotics for the treatment of traveler's diarrhea.

A meta-analysis^7^ conducted by McFarland showed that probiotics significantly prevented traveler's diarrhea. The author included 12 studies (n = 4079) in the analysis and observed a reduced relative risk for diarrhea in travelers who used probiotics (RR = 0.85; 95% CI, 0.79–0.91; *P* < 0.001). In a study by Katelaris et al, the ingestion of probiotics did not reduce the frequency of diarrhea among 282 British soldiers who were deployed to Belize.^8^ A study by Hilton et al randomized 245 American tourists who were traveling to various developing countries to receive either *L rhamnosus* GG (LGG) or a placebo. The travelers who ingested LGG exhibited a mean daily risk of developing diarrhea of 3.9%, compared with 7.4% in the travelers who received the placebo (*P* = 0.05).^9^

A Cochrane review including 23 studies revealed that probiotics are effective in reducing the risk of diarrhea (RR = 0.66; 95% CI, 0.55–0.77; *P* = 0.02) and duration of diarrhea (95% CI, 18.51–42.46 h; *P* < 0.00001). The authors agreed that probiotics are useful in conjunction with rehydration therapy in treating acute infectious diarrhea. However, additional research is needed to establish clear guidelines for providers.^10^

In an analysis of data from 34 blinded, randomized, placebo-controlled trials in a meta-analysis, Sazwan et al concluded that the use of probiotics in antibiotic-associated diarrhea decreased the risk of diarrhea by 52% (95% CI, 35–65%), traveler's diarrhea by 8% (95% CI, −6 to 21%), and acute diarrhea from various causes by 34% (8–53%).^11^ Probiotics were more effective in reducing the risk of acute diarrhea in children 57% (95% CI, 35–71%) versus 26% (95% CI, 7–49%) in adults.^11^

### Antibiotic-Associated Diarrhea

Studies have demonstrated that probiotics reduce the frequency of antibiotic-associated diarrhea. Several meta-analyses have shown positive results for the use of probiotics in antibiotic-associated diarrhea. Cremonini et al examined 7 randomized controlled trials (n = 881) and concluded that the use of probiotics was beneficial for the treatment of antibiotic-associated diarrhea (RR = 0.396; 95% CI, 0.27–0.57).^12^ Another meta-analysis by McFarland included 25 randomized controlled trials (n = 2810) and revealed significant reductions in diarrheal episodes that were associated with antibiotics.^13^ However, not all probiotic strains are effective under all conditions. A meta-analysis by McFarland in hospitalized adult patients showed that LGG does not reduce the frequency of diarrheal episodes.^13^ Another study demonstrated that the use of a combination of products that included *L casei*, *L bulgaricus*, and *S thermophilus* twice daily for 1 week decreased additional cases of diarrhea.^14^ One of the problem observed in hospitalized adult patients who have been treated with antibiotics is Clostridium difficile infection. A meta-analysis reviewing 6 randomized controlled trials revealed that only *S boulardii* in combination with oral metronidazole and/or vancomycin significantly decreased *C difficile* infection.^13^ A Cochrane review also found that there was insufficient evidence to support the use of probiotics in *C difficile* infections.^15^

### Acute Diarrhea in Children

Rotavirus is the most common virus that is associated with acute diarrhea in children aged 1 month to 3 years. A double-blind, placebo-controlled study by the European Society for Pediatric Gastroenterology, Hepatology, and Nutrition involving 287 children aged 1 to 36 months concluded that probiotics are more effective than a placebo or hydration alone for treating acute diarrhea^16^ (58.3 ± 27.6 h vs 71.9 ± 35.8 h). Another meta-analysis^17^ evaluated the efficacy of probiotic therapy in acute infectious diarrhea of varying etiologies, including rotavirus in children; the author concluded that probiotics are effective in reducing the duration of diarrhea by 0.7 days. Two studies by Shornikova et al^18,19^ showed similar results. Another meta-analysis^20^ included 18 studies that involved children who were under 5 years of age with acute diarrhea and received probiotics with standard rehydration therapy. The studies in this meta-analysis involved hospitalized children and were double blinded, controlled trials. The authors concluded that the use of probiotics with rehydration therapy reduces the duration of acute diarrhea by 1 day (95% CI, –1.1 to –0.6; *P* < 0.001).

A Cochrane review^21^ analyzed 63 trials (n = 8014) that mainly involved infants and children. According to the author, all of the studies reported a shortened duration of diarrhea and reduced stool frequency. The study concluded that there are beneficial effects of probiotics for the treatment of acute diarrhea. However, this review suggested that further studies are required to establish clear guidelines for product selection, dosing, and specific patient populations.

### Irritable Bowel Syndrome (IBS)

The use of probiotics in IBS is not well established. Some studies have shown an improvement of some symptoms of IBS after probiotics use; however, definitive evidence is lacking. A meta-analysis by McFarland^22^ reviewed 20 trials that included a total of 1404 patients. These studies were randomized, controlled, and blinded trials that investigated the use of probiotics for the treatment of IBS. This meta-analysis revealed that probiotic use was associated with an improvement in global IBS symptoms compared with placebo (RR_pooled_ = 0.78; 95% CI, 0.62–0.96). Additionally, there was a decrease in IBS-associated pain. This study did not evaluate other symptoms, such as bloating, diarrhea, or flatulence. The author briefly discussed the beneficial effects of probiotics in IBS but suggested a cautious interpretation of these effects, as sufficient data were lacking based on these studies.

In a randomized blinded study,^23^ 50 adults aged 26 to 64 years who were diagnosed with IBS according to the Rome 11 criteria were randomly selected to receive either probiotics containing *L plantarum* and *B breve* at a concentration of 5 × 10^10^ CFU/mL or placebo. After 14 days of treatment, pain was decreased by 38% in the probiotic group compared with 18% in the placebo group (*P* < 0.05). Furthermore, after 28 days, pain was decreased 52% in the probiotic group compared with 11% in the placebo group (*P* < 0.001). The study concluded that there are beneficial effects of probiotic use in IBS.

In a clinical study, a combination product designated VSL#3, which contains large quantities of 8 lyophilized bacterial species, was shown to significantly improves IBS symptoms.^24^ However, another clinical trial using LGG tablets did not reveal any significant improvement in IBS symptoms.^25^

### Inflammatory Bowel Disease (IBD)

IBD is a combination of diseases that includes Crohn's disease, ulcerative colitis (UC), and pouchitis. The exact etiology of IBD is unknown; however, a change in the normal intestinal flora is one of the manifestations of this disease. The mechanism of action of probiotics in IBD is not fully understood. Probiotics may exert their effects by modulating the intestinal flora^26^ and the intestinal immune response.^27^

A clinical review published by Ailsa et al in the Journal of Gastroenterology discussed the efficacy of probiotics for the treatment of IBD. The authors demonstrated the efficacy of probiotics in IBD but suggested that there is a need for a large controlled trial to determine definitive criteria concerning the use of probiotics in IBD treatment.^28^ Another review demonstrated the efficacy of probiotics in pouchitis and ulcerative colitis but found that evidence was lacking for the use of probiotics in Crohn's disease.^29,30^

A randomized double-blind study^31^ showed that the probiotic *E coli* Nissle 1917 is as effective as mesalazine at maintaining remission in patients with ulcerative colitis. This study included 327 patients who participated in a double-blind trial and were assigned to receive either the probiotic drug (n = 162) once daily or mesalazine (n = 165) at 500 mg 3 times daily. After a 12-month period, the patients were assessed clinically, endoscopically, and histologically. The study revealed that relapses occurred in 40 of the 110 (36.4%) patients in the *E coli* Nissle 1917 group and 38 out of 112 (33.9%) in the mesalazine group (*P* = 0.003).

A Cochran review concluded that adding probiotics to standard therapy may provide some benefits; however, the supporting data are limited. For Crohn's disease, there is insufficient evidence to draw any conclusions about the effectiveness of probiotics.^32,33^

### Atopic Dermatitis

The exact mechanism of action of probiotics for the treatment of atopic dermatitis is not fully understood. The mechanism is believed to involve immunomodulatory effects. Studies have shown that the use of LGG in infants with atopic dermatitis may be beneficial. A double-blind, placebo-controlled study involving 62 pregnant women who had a strong family history of atopic dermatitis randomized these subjects to receive either LGG or a placebo during the last weeks of their pregnancy and during breastfeeding. The study revealed that the infants of the mothers who had received LGG presented a significantly lower risk of developing atopic dermatitis during the first 2 years of life.^38^

Another double-blind, randomized study^39^ was conducted on 27 infants aged between 4 and 6 months who were diagnosed with eczema. These infants received either formula with probiotics (LGG or B lactis) or the same formula without probiotics. After 2 months, eczema symptoms were significantly improved in the probiotics group compared with the group that did not receive probiotics (*P* = 0.002).

A Cochran review including 12 randomized controlled trials involving 781 children found no significant difference between groups that received probiotics versus placebo. The authors concluded that there was insufficient evidence to recommend the use of probiotics for the treatment of eczema.^40^

### Safety and Side Effects of Probiotics

Most probiotics are safe. However, care should be taken when administering probiotics to severely ill or immunocompromised patients. There have been rare incidents of sepsis, endocarditis, and liver abscess during the use of Lactobacillus; additionally, fungemia has been reported with the use of *S boulardii*, primarily in patients with severe comorbidities.^41,42^

The most common side effects of probiotics are constipation, flatulence, hiccups, nausea, infection, and rash.

## CONCLUSION

Probiotics are generally safe; however, they should not be used in critically ill or immune-compromised patients, and their use during pregnancy and in infants should be cautioned. The risks and benefits of probiotics should be weighed before their use. Probiotics appeared to exert some beneficial effects in GI-related illnesses. The use of probiotics in non-GI illnesses is not sufficiently supported by current data.
